# Serum levels of 25-hydroxy vitamin D correlate with idiopathic benign paroxysmal positional vertigo

**DOI:** 10.1042/BSR20190142

**Published:** 2019-04-30

**Authors:** Jing Ding, Lei Liu, Wei-Kuan Kong, Xiao-Bing Chen, Xudong Liu

**Affiliations:** 1 Department of Neurology, Hanting District People’s Hospital, Weifang, China; 2 Department of Pain, Qian Foshan Attached Hospital of Shandong University, Jinan, China; 3 Department of Pain, Liaocheng Second People’s Hospital, Liaocheng, China; 4 Department of Orthopedics, Zhangqiu City Hospital of Traditional Chinese Medicine, Jinan, China; 5 Department of Pain, Qilu Hospital of Shandong University, Jinan, China

**Keywords:** 25-hydroxy vitamin D, benign paroxysmal positional vertigo, recurrence, vitamin D deficiency

## Abstract

**Background:** The present study aimed to evaluate serum 25-hydroxy vitamin D (25(OH) D) levels in Chinese patients with idiopathic benign paroxysmal positional vertigo (BPPV) and to investigate the possible relationship between the occurrence and recurrence of idiopathic BPPV and low 25(OH) D levels. **Methods**: Between 1 January 2017 and 31 May, 2018, consecutively older patients (age, older than 50 years) with idiopathic BPPV were recruited in the present study. For each patient, 2:1 sex and age matched healthy people were assigned as the control group. The influence of 25(OH) D levels on BPPV and recurrent BPPV were performed by binary logistic regression analysis. **Results:** In the present study, 174 patients with BPPV and 348 controls were included. The serum levels of 25(OH) D in those patients were lower than in those controls (*P*<0.001). One hundred eight patients were found to have vitamin D deficiency; thus, the prevalence was 62.1%, which was higher than that in the controls (42.8%). The data showed that patients with recurrent BPPV (N = 31) had lower serum levels of 25(OH) D compared with those who were not (11.2 ng/ml [interquartile range, 7.2–20.8 ng/ml] vs 18.7 ng/ml [14.2–24.8 ng/ml]). The regression analyses demonstrated that vitamin D deficiency was associated with BPPV and recurrent BPPV with an odds ratio of 2.15 (95% confidence interval [CI], 1.30–4.32; *P*=0.006) and 5.16 (95% CI, 1.00–34.12; *P*=0.05). **Conclusion:** Decreased serum levels of 25(OH)D were associated with the occurrence and recurrence of BPPV in a Chinese population, independent of other baseline markers.

## Introduction

Benign paroxysmal positional vertigo (BPPV) is the most commonly diagnosed type of vertigo and is characterized by short-duration vertigo, nausea, and/or positional nystagmus associated with changes in head position [1]. This condition presented as dizziness or vertigo of sudden onset that is provoked by certain changes in head position [2]. A previous epidemiology study found that the lifetime prevalence of BPPV was 2.4%, the 1-year prevalence was 1.6%, and the 1-year incidence was 0.6% [3]. However, only 8% of the affected participants had obtained effective treatment [3].

BPPV can cause severe impact, including reduced daily activities, falls, and depression on the quality of life, especially in elderly patients [4]. Patients with BPPV exhibited a 1.14-fold (95% confidence interval [CI], 1.04–1.25; *P*<0.01) risk of fracture than those without BPPV [5]. BPPV is thought to be caused by the presence of cupulolithiasis or canalithiasis in one or more semicircular canals. However, the exact etiology is unknown [1]. Calcium metabolism plays a primary role in the synthesis/absorption of otoconia made of calcium carbonate and thus might be an etiological factor in the onset of BPPV. Several studies indicated the association between BPPV with osteoporosis and vitamin D deficiency, implying that abnormal calcium metabolism may underlie BPPV [6].

A previous study found that the mean serum 25-hydroxy vitamin D (25(OH) D) levels were also significantly lower in female patients with BPPV than in healthy controls (*P*<0.001) [7]. Several studies found a correlation between vitamin D deficiency and the development, and the recurrence of BPPV [6, 8–9]. They suggested that vitamin D deficiency results in the production of abnormal otoconia, which results in otolith dysfunction [10]. Nevertheless, a few recent studies reported that a low vitamin D level was not associated with BPPV occurrence and/or recurrence [11–13]. Considering these inconsistencies, the relationship between BPPV and vitamin D deficiency is debatable.

The aim of the present study is to evaluate serum 25 (OH)D levels in Chinese patients with idiopathic BPPV and to investigate the possible relationship between the occurrence and recurrence of BPPV and low 25(OH) D levels.

## Patients and Methods

Between 1 January 2017, and 31 May 2018, consecutively older patients (age, older than 50 years) with idiopathic BPPV at the Department of Neurology of Lanzhou General Hospital of PLA, China we recruited in the present study. The study was performed at geographic latitude 36.03°N. The BPPV diagnosis was based on a characteristic history and observation of typical nystagmus during the Dix-Hallpike maneuver, supine roll, and cephalic hyperextension tests [7]. A typical history of brief attacks of positional vertigo was obtained from all patients with BPPV in whom the apparent etiology was absent and described as idiopathic [14].

The exclusion criteria included (1) noncooperation; (2) having secondary factors for BPPV, such as a history of head trauma, vestibular neuritis, Meniere’s disease, migraines, ear surgery or sudden hearing loss, having a hip or lumbar spine fracture; (3) malignant, chronic renal, hepatic, stroke, cardiovascular or autoimmune diseases, gout, hypothyroidism or hyperthyroidism, the use of any drug, in particular allopurinol and/or diuretics, and a history of neurological diseases. The patients used vitamin and/or calcium supplements, and patients with systemic diseases influencing vitamin D levels during the study were excluded.

For each patient, 2:1 sex- and age-matched healthy people from the medical center at our hospital were assigned as the control group. The study protocol was approved by the Qilu Hospital of Shandong University Research Ethics Committee, and written informed consents were obtained from all included cases.

For each included case, age, sex, body mass index (BMI), blood pressure, the seasons into study, regular physical activity habits (walking at a brisk pace for 30 min or more, three times a week) and medication status (diabetes mellitus, hypertension, hyperlipidemia, smoking, drinking) were recorded. BMI was calculated as weight in kilograms divided by the square of height in meters. The intensity of BPPV was assessed by the patients and was expressed as visual analogue scale (VAS) score (0–10), where 0 indicated no vertigo and 10 indicated severe attacks of vertigo [15]. The posterior semicircular canal (PSC) type of BPPV and the horizontal semicircular canal (HSC) were diagnosed [4]. The patients were divided into two groups according to the recurrence or nonrecurrence of BPPV. Recurrent BPPV was defined when the patients reported two or more previous episodes of positional vertigo similar to those experienced at the time of diagnosis, with at least 1-month interval [16].

The serum samples were prospectively drawn from the antecubital vein at the first morning after admission. After centrifugation, the samples were immediately stored at −80°C before assay. Serum level of 25(OH) D was measured with competitive chemiluminescent immunoassay in a calibrated Elecsys 2010 (Roche diagnostics GmbH, Germany).

### Statistical analysis

Results were expressed as percentages for categorical variables and as medians (interquartile ranges, [IQRs]). Univariate data on demographic and clinical features were compared by Mann–Whitney *U*-test or χ^2^ test as appropriate. Correlations among continuous variables were assessed by the Spearman rank-correlation coefficient.

The influence of 25(OH) D levels on BPPV and recurrent BPPV were performed by binary logistic regression analysis, which allows adjustment for possible confounding factors (age, sex, BMI, season, diabetes mellitus, hypertension, hyperlipidemia, smoking, drinking, VAS score, regular exercise habit, and different semicircular canals). Results were expressed as adjusted odds ratios (OR) with the corresponding 95% CI. In addition, multivariate analysis models were applied to further explore the relation of BPPV and vitamin D state. The 25(OH) D levels are therefore used to classify the vitamin D status into vitamin D deficiency (<20 ng/ml), vitamin D insufficiency (20–29 ng/ml) and vitamin D sufficiency (≥30 ng/ml) [17]. We also conducted subgroup analyses separately among men and women cases.

Receiver operating characteristic (ROC) curves were utilized to evaluate the accuracy of serum 25(OH) D to predict BPPV. Area under the curve (AUC) was calculated as measurements of the accuracy of the test. All statistical analysis was performed with SPSS for Windows, version 20.0 (SPSS Inc., Chicago, IL, U.S.A.). Statistical significance was defined as *P*<0.05.

## Results

### Baseline clinical characteristics

**Figure 1:**
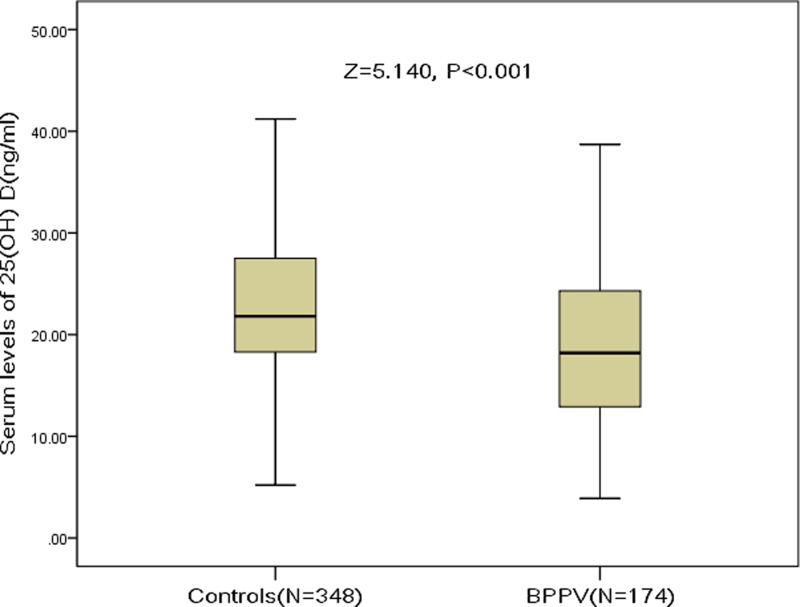
Comparisons of serum 25(OH) D levels between patients with BPPV and controls Mann–Whitney *U*-test. All data are medians and IQR.

In the present study, 174 patients with BPPV and 348 controls were included. The serum levels of 25(OH) D in those patients were lower than those controls (18.2 ng/ml [IQR, 12.7–24.3 ng/ml] vs 21.8 ng/ml [IQR, 18.3–27.6 ng/ml]; *P*<0.001; Figure 1). One hundred eight patients were diagnosed to have vitamin D deficiency; thus, the prevalence was 62.1%, which was higher than that in the controls (42.8%) (Table 1). Most (83.3%) of the patients were evaluated within 2 weeks from symptom onset. Baseline clinical characteristics of patients and controls are listed in Table 1.

**Table 1:** Characteristics of patients and controls

	BPPV	Controls	*P*^‡^
N	174	348	
Age, median (IQR), years	61 (54–69)	61 (54–69)	NS
Sex-female, n (%)	102 (58.6)	204 (58.6)	NS
BMI, median (IQR), kg/m^2^	25.8 (24.3–27.4)	26.0 (24.4–27.6)	NS
Systolic blood pressure, median (IQR), mmHg	125 (115–135)	123 (114–130)	NS
Diastolic blood pressure, median (IQR), mmHg	80 (75–86)	78 (72–85)	NS
Including season-winter, n (%)	51 (29.3)	105 (30.2)	NS
Diabetes mellitus, n (%)	25 (14.4)	55 (15.8)	NS
Hypertension, n (%)	31 (17.8)	66 (19.0)	NS
Hyperlipidemia, n (%)	44 (25.3)	94 (27.0)	NS
Smoking, n (%)	28 (16.1)	53 (15.2)	NS
Drinking, n (%)	19 (10.9)	35 (10.1)	NS
Regular exercise habit, n (%)	21 (12.1)	40 (11.5)	NS
VAS score median (IQR)	4 (1–6)	–	
25(OH) D, ng/ml	18.2 (12.7–24.3)	21.8 (18.3–27.6)	<0.001
Vitamin D deficiency, n (%)^†^	108 (62.1)	149 (42.8)	<0.001
Vitamin D insufficiency, n (%)^†^	42 (24.1)	132 (37.9)	0.002
Vitamin D sufficiency, n (%)^†^	24 (13.8)	67 (19.3)	0.12
Different semicircular canals, n (%)		–	
Posterior	122 (70.1)		
Horizontal	45 (25.9)		
Anterior	7 (4.0)		

‡ the *P-*value was tested by Mann–Whitney *U*-test or χ^2^ test.

† The 25(OH) D levels are therefore used to classify the vitamin D status into vitamin D deficiency (<20 ng/ml), vitamin D insufficiency (20–29 ng/ml) and vitamin D sufficiency (≥ 30 ng/ml).

**Abbreviation**: DM: diabetes mellitus.

### Characteristics of BPPV

BPPV most commonly involved the posterior canal (*n*=122, 70.1%), followed by the horizontal (*n*=45, 25.9%) and anterior canal (*n*=7, 4.0%). The 25(OH) D levels did not differ statistically in patients with different semicircular canals (*P*>0.05). With respect to the most frequent involvement (PSC), the right side was affected in 72 (59.0%) patients and the left side in 50 (41.0%) patients. The 25(OH) D levels did not differ statistically in patients with PSC BPPV for either the right or left side (*P*>0.05). The median score of VAS was 4 (IQR, 1–6). As a continuous variable, it was found that there was a correlation between VAS score and serum levels of 25(OH) D (r = −0.348; *P*<0.001). Recurrent attacks of BPPV were reported in 31 patients.

### Serum 25(OH) D levels and risk of BPPV

As a continuous variable, 25(OH) D was associated with the decreased risk of BPPV (OR, 0.96; 95% CI, 0.94–0.98; *P*=0.006) in the univariate model. Multiple-logistic regression analyses adjusted for age, sex, BMI, season, diabetes mellitus, hypertension, hyperlipidemia, smoking, drinking, and regular exercise habit suggested that 25(OH) D was still associated with the decreased risk of BPPV (OR, 0.98; 95% CI, 0.96–0.99; *P*=0.009). In addition, as a categorical variable, the results demonstrated that vitamin D deficiency was associated with BPPV (OR, 2.15; 95% CI, 1.30–4.32, *P*=0.006; Table 2).

**Table 2:** Univariate and multivariate analyses for BPPV according to vitamin D state

Vitamin D state^†^	BPPV/All^††^	Crude OR (95% CI), *P*^#^	Multivariable-adjusted OR^‡^, *P*^#^
Vitamin D deficiency	108/232	2.43 (1.43–4.14), 0.001	2.15 (1.30–4.32), 0.006
Vitamin D insufficiency	42/199	0.75 (0.42–1.33), 0.32	0.70 (0.37–1.55), 0.48
Vitamin D sufficiency	24/91	Reference	Reference

† The 25(OH) D levels are therefore used to classify the vitamin D status into vitamin D deficiency (<20 ng/ml), vitamin D insufficiency (20–29 ng/ml) and vitamin D sufficiency (≥30 ng/ml).

†† All included BPPV and controls.

‡ Adjusted for factors including age, sex, BMI, season, diabetes mellitus, hypertension, hyperlipidemia, smoking, drinking, and regular exercise habit.

# *P*-value for the trend <0.001.

**Figure 2:**
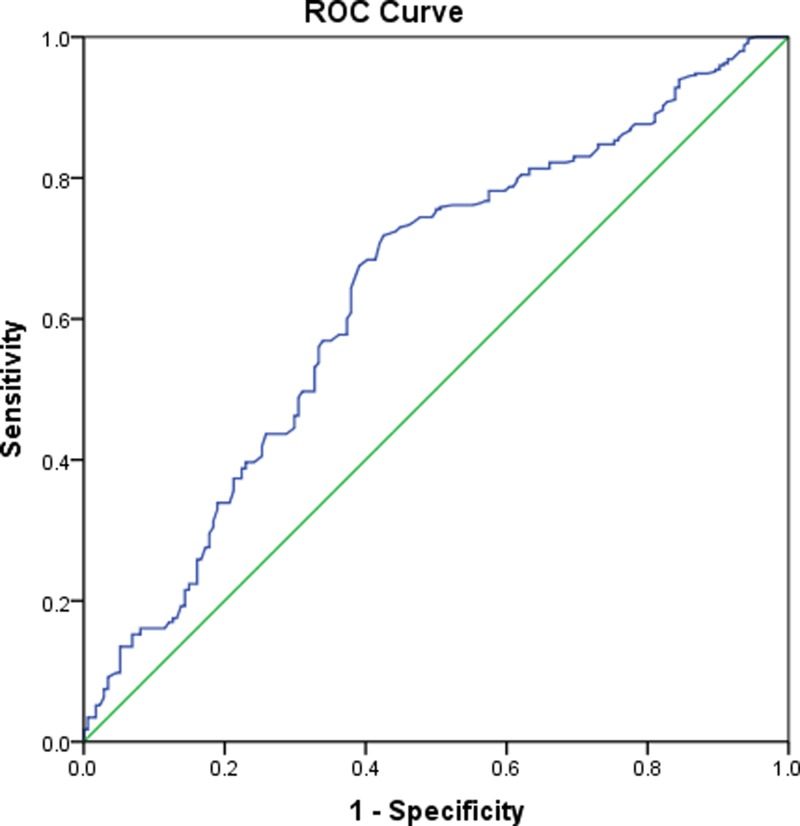
Receiver operator characteristic curve demonstrating sensitivity as a function of 1-specificity for predicting the BPPV based on serum level of 25(OH) D

Based on the ROC curve, the projected optimal cut-off value of serum 25(OH) D levels as an indicator for the diagnosis of BPPV was 16.8 ng/ml, which yielded a sensitivity of 71.5% and a specificity of 64.5%, with an AUC of 0.64 (95% CI, 0.59–0.69). See Figure 2.

### Serum 25(OH) D levels and risk of recurrent BPPV

**Figure 3:**
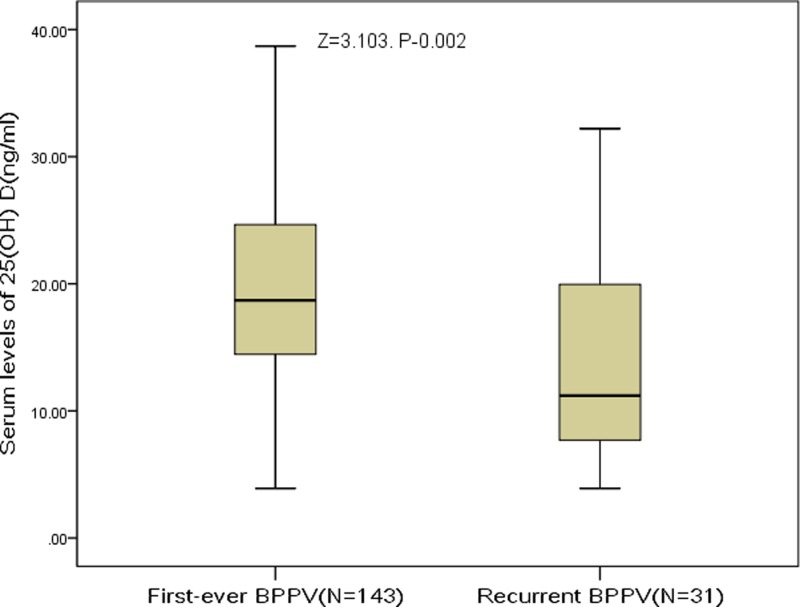
Comparisons of serum 25(OH) D levels between patients with recurrent BPPV and *de novo* BPPV Mann–Whitney *U*-test. All data are medians and IQR.

The patients were divided into two groups according to the recurrence or nonrecurrence of BPPV. Figure 3 showed that patients with recurrent BPPV had lower serum levels of 25(OH) D compared with nonrecurrent BPPV (11.2 ng/ml; IQR, 7.2–20.8 ng/ml vs 18.7 ng/ml; 14.2–24.8 ng/ml). As a continuous variable, 25(OH) D was associated with decreased risk of recurrent BPPV (OR, 0.92; 95% CI, 0.87–0.97; *P*=0.002) in the univariate model. Multiple-logistic regression analyses adjusted for age, sex, BMI, season, diabetes mellitus, hypertension, hyperlipidemia, smoking, drinking, VAS score, regular exercise habit, and different semicircular canals showed that 25(OH) D was still associated with decreased risk of recurrent BPPV (OR, 0.94; 95% CI, 0.90–0.98; *P*=0.006). In addition, as a categorical variable, the results suggested that vitamin D deficiency was associated with recurrent BPPV with the ORs of 5.16 (95% CI, 1.00–34.12; *P*=0.05) (Table 3).

**Table 3:** Univariate and multivariate analyses for recurrent BPPV according to vitamin D state

Vitamin D state ^†^	RBPPV/BPPV	Crude OR (95% CI), *P*^#^	Multivariable-adjusted OR^‡^, *P*^#^
Vitamin D deficiency	25/108	6.93 (1.03–33.12), 0.03	5.16 (1.00–34.12), 0.05
Vitamin D insufficiency	5/42	3.11 (0.34–28.31), 0.29	2.85 (0.36–28.55), 0.42
Vitamin D sufficiency	1/24	Reference	Reference

† The 25(OH) D levels are therefore used to classify the vitamin D status into vitamin D deficiency (<20 ng/ml), vitamin D insufficiency (20–29 ng/ml) and vitamin D sufficiency (≥30 ng/ml).

‡ Adjusted for factors including Age, sex, BMI, season, diabetes mellitus, hypertension, hyperlipidemia, smoking, drinking, VAS score, regular exercise habit, and different semicircular canals.

# *P*-value for the trend <0.001.

**Abbreviations**: RBPPV, recurrent BPPV.

**Figure 4:**
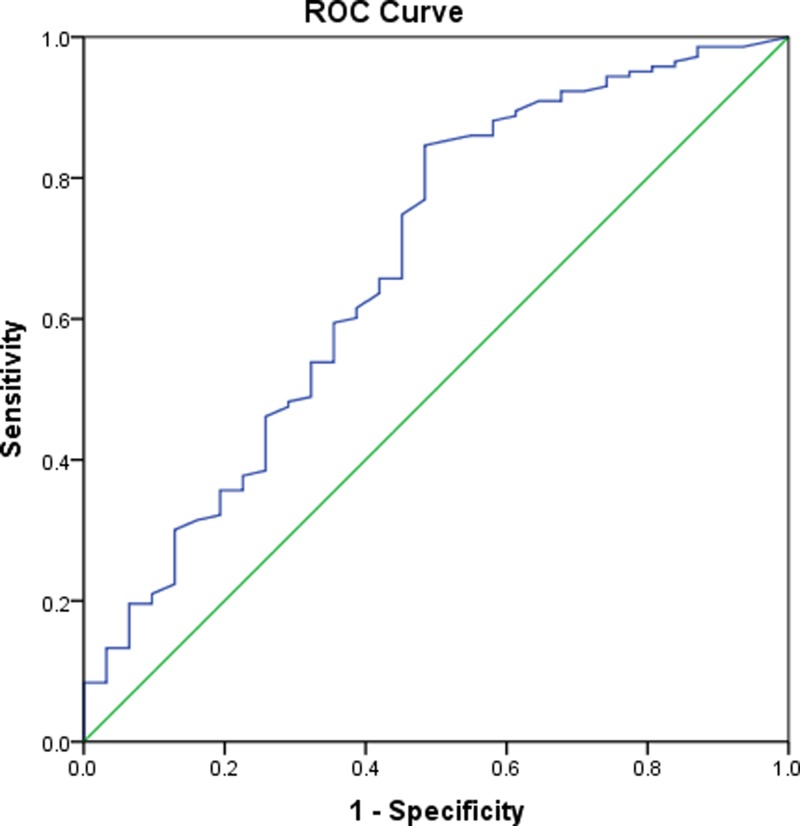
Receiver operator characteristic curve demonstrating sensitivity as a function of 1-specificity for predicting the recurrent BPPV based on serum level of 25(OH) D

Based on the ROC curve, the optimal cut-off value of serum 25(OH) D levels as an indicator for diagnosis of BPPV was projected to be 14.2 ng/ml, which yielded a sensitivity of 84.5% and a specificity of 57.4%, with an AUC of 0.68 (95% CI, 0.58–0.79). See Figure 4.

### Sub-group analysis

Furthermore, we also conducted analyses separately among male and female cases. In the multivariate regression analysis, the date demonstrated that for each 1 ng/ml increase of serum concentration of 25(OH) D, the association with risk of BPPV was stronger among female cases (OR, 0.95; 95% CI, 0.93–0.97; *P*=0.001) versus male cases (OR, 0.98; 95% CI, 0.96–0.99; *P*=0.012). Interestingly, the predictive value of 25(OH) D to predict recurrent BPPV (OR, 0.92; 95% CI, 0.89–0.97; *P*=0.002) was also stronger in female cases than in male cases (0.95 [0.92–0.99]; *P*=0.015).

## Discussion

Research on the relationship between vitamin D deficiency and vertigo is scarce, and this context has not been adequately investigated. In the present study, we evaluated serum 25 (OH)D levels in Chinese patients with idiopathic BPPV and to investigate the possible relationship between the occurrence and recurrence of BPPV and low 25(OH) D levels. The present study clarified that (1) serum levels of 25(OH) D were lower in BPPV than that in controls; (2) serum levels of 25(OH) D were lower in the recurrence of BPPV than in the *de novo* BPPV; (3) the regression analyses demonstrated that vitamin D deficiency was associated with BPPV and recurrent BPPV with an OR of 2.15 (95% CI, 1.30–4.32; *P*=0.006) and 5.16 (95% CI, 1.00–34.12; *P*=0.05), suggesting that low 25(OH) D may be a risk factor for BPPV and recurrent BPPV; (4) this correlation between vitamin D deficiency wand BPPV was stronger in women than in men; (5) serum levels of 25(OH) D were negative associated with intensity of BPPV (assessed by VAS score).

Consistent with our finding, one study demonstrated that vitamin D deficiency was associated with BPPV with an OR of 2.1 (95% CI, 1.1–3.1; *P*=0.031) in postmenopausal women [7], another study showed that vitamin D deficiency was associated with BPPV with an odds ratio of 2.054 (95% CI, 1.088–3.877; *P*=0.026) in female patients [18]. Similarly, Talaat et al. [6] found that low levels of vitamin D were related to development of BPPV, whereas very low levels were associated with recurrence of BPPV. Furthermore, Jeong et al. [8] reported that decreased vitamin D only showed a significant relationship with BPPV occurrence, but the vitamin D level did not differ between the *de novo* and recurrent groups. Yang et al. [19] showed that serum 25(OH) D levels in men were associated with the occurrence of BPPV. Furthermore, a recent meta-analysis study showed that low vitamin D level was significantly evident among patients with recurrent episodes of BPPV; however, there was a failure in establishing a relationship between the occurrence of BPPV and low vitamin D level [20]. These differences might be caused by different clinical settings. In addition, serum 25(OH) D levels were also affected by various factors, such as age, sex, seasonal factors, hormonal factors, location, nutrition and lifestyle habits, and preexisting metabolic disorders [21].

The relationship between decreasing levels of 25(OH) D and BPPV was reported in the present study. A more meaningful study was whether vitamin supplementation can improve the BPPV, especially for BPPV patients with vitamin D deficiency. However, our cross-sectional design could not obtain any causal relation. One study showed that the treatment of BPPV with 1α-hydroxyvitamin D3 could effectively improve the symptoms of the patients, and the level of vitamin D3 and the occurrence of osteopenia/osteoporosis were the clinical indexes of whether the BPPV treatment was effective [22]. Another study indicated that the normalization of serum vitamin D significantly reduces BPPV recurrences [23]. Similarly, the recurrences of BPPV were improved after correction of vitamin D deficiency [24]. The beneficial effect of vitamin D therapy on severity of BPPV may be attributed to direct the effect of vitamin D on vestibular system or indirect effect of vitamin D, on muscle strength, fall, balance, and musculoskeletal system [25–27]. The establishment of the causal link between BPPV and decreased vitamin would require animal experiments and eventually a clinical trial that looks for preventive effects of vitamin D supplementation on recurrence of BPPV in patients with BPPV and low serum vitamin D.

The relationship between decreasing levels of 25(OH) D and BPPV reported in the present study was no proof of a causal relation due to the cross-sectional design; however, there are mechanisms through which low levels of 25(OH) D could have harmful effects on the BPPV. First, the epithelial Ca2^+^ channel transport system, Na^+^/Ca2^+^ exchangers, and plasma membrane Ca2^+^ pumps expressed in the inner ear contribute to this critical balance of calcium levels by transepithelial absorption of Ca2^+^ from the endolymph of the inner ear [28]. Vitamin D regulates the expression of some Ca2^+^ binding proteins via vitamin D receptors (VDR) in the epithelial cells of the inner ear [28]. Therefore, it has been speculated that vitamin D deficiency also contributes to the development of BPPV by abnormal calcium metabolism in the inner ear [19]. Second, VDR-deficient mice showed a balance dysfunction [29]. Previous studies found VDR in epithelial cell of crista ampullaris, membranous semicircular canals, and surrounding bone cells in mice [22]. In addition, the accelerated rotation, inclined platform, rotation, and swimming tests show that the equilibrium function of mice with mutant VDR is decreased, suggesting that vitamin D insufficiency may cause the vestibular dysfunction, such as BPPV [29–30]. Subjects with vitamin D deficiency have abnormal ocular and cervical vestibular evoked myogenic potentials, indicating that vitamin D deficiency results in otolith dysfunction [10].

One limitation of the present study was the relatively small sample size (N = 174), which made it impossible to draw firm conclusions in the sub-group analysis. Especially, the recurrent case was so small (N = 31), and the multivariate analysis using these data may have some problem. For the generalization of the results, the sample size should be larger. Second, we only tested the serum levels of 25(OH) D once time and did not perform follow-up to detect the values of 25(OH) D at different stages of the disease (e.g., acute stage or remission stage). Third, because our study was cross-sectional, we were unable to establish a causal relationship between 25(OH) D and the development of BPPV. In addition, we evaluated only idiopathic BPPV. We were unable to identify the association between comorbidities and BPPV, both of which are affected by aging. Last, because of some technical limitations, we did not test the biomarkers of osteoporosis and VDR, which should be considered in future studies.

## Conclusion

In summary, decreased serum levels of 25(OH)D were associated with the occurrence and recurrence of BPPV in a China population, independent of other baseline markers. The measurement of serum levels of vitamin D might be helpful in diagnosing and managing BPPV patients. Because it plays an important role at multiple stages of BPPV progression, vitamin D will be worthy of further research as a possible therapeutic target.

## Ethics, consent, and permissions

Written informed consents were obtained from all patients; and, the present study conformed to the principles of the Declaration of Helsinki was approved by the investigational review board of the Qilu Hospital of Shandong University.

## Data Availability

Please contact corresponding author for data requests.

## Abbreviations

25(OH) D: 25-hydroxy vitamin D
AUC: area under the curve
BMI: body mass index
CI: confidence interval
BPVV: benign paroxysmal positional vertigo
OR: odd ratio
PSC: posterior semicircular canal
ROC: receiver operating characteristic
VAS: visual analogue scale
VDR: vitamin D receptor
